# Computing Excited States of Molecules Using Normalizing Flows

**DOI:** 10.1021/acs.jctc.5c00590

**Published:** 2025-05-15

**Authors:** Yahya Saleh, Álvaro Fernández Corral, Emil Vogt, Armin Iske, Jochen Küpper, Andrey Yachmenev

**Affiliations:** † Department of Mathematics, Universität Hamburg, Bundesstr. 55, 20146 Hamburg, Germany; ‡ Deutsches Elektronen-Synchrotron DESY, 155358 Center for Free-Electron Laser Science CFEL, Notkestr. 85, 22607 Hamburg, Germany; § Department of Physics, 14915 Universität Hamburg, Luruper Chaussee 149, 22761 Hamburg, Germany; ∥ Center for Ultrafast Imaging, Universität Hamburg, Luruper Chaussee 149, 22761 Hamburg, Germany

## Abstract

Calculations of highly excited and delocalized molecular vibrational states are computationally challenging tasks, which strongly depend on the choice of coordinates for describing vibrational motions. We introduce a new method that leverages normalizing flows, i.e, parametrized invertible functions, to learn optimal vibrational coordinates that satisfy the variational principle. This approach produces coordinates tailored to the vibrational problem at hand, significantly increasing the accuracy and enhancing the basis set convergence of the calculated energy spectrum. The efficiency of the method is demonstrated in calculations of the 100 lowest excited vibrational states of H_2_S, H_2_CO, and HCN/HNC. The method effectively captures the essential vibrational behavior of molecules by enhancing the separability of the Hamiltonian and hence allows for an effective assignment of approximate quantum numbers. We demonstrate that the optimized coordinates are transferable across different levels of basis set truncation, enabling a cost-efficient protocol for computing vibrational spectra of high-dimensional systems.

## Graphical abstract

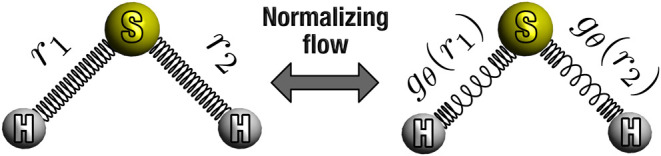

## I Introduction

Accurate calculations of highly excited vibrational states of polyatomic molecules are essential for unraveling increasingly rich experimental spectroscopic information and understanding the dynamics of intermolecular motions. The highly excited molecular vibrations are especially important in fields such as chemical reactivity^1–3^ and collisions,^4,5^ relaxation processes,^6^ and stimulated emission,^7^ as well as spectroscopic probing of high-temperature environments found on exoplanets^8,9^ and in industrial applications.^10,11^

A range of variational and perturbative methods were developed for predicting vibrational spectra of molecules.^12–18^ These methods solve the eigenvalue problem for a vibrational Hamiltonian, which is constructed using appropriately chosen vibrational coordinates. The choice of coordinates is a crucial task that directly affects the accuracy of the energy calculations. When using a direct product basis of univariate functions, a key challenge is selecting coordinates that provide a large degree of separability of vibrational motions, thereby reducing the computational effort required to solve the vibrational eigenvalue problem.^19^ This is particularly important for calculations of delocalized vibrational states of floppy molecules,^20,21^ such as van der Waals complexes,^22^ molecules near dissociation,^23^ or high-energy excitations in general,^24^ where couplings between different vibrational modes are prominent.

Rectilinear normal coordinates provide a natural starting point for seeking separability in vibrational problems. However, they become less effective for highly excited states and are generally not suited for floppy molecules, e.g., weakly bound complexes, which naturally sample configurations far from their reference equilibrium geometry. Alternative curvilinear coordinate systems, such as Radau,^25^ Jacobi,^26,27^ valence,^28^ and polyspherical^29–31^ coordinates, were successfully applied in the vibrational calculations of various floppy polyatomic molecules.^13,32^ Choosing the optimal coordinates requires a combination of intuition, consideration of the symmetries of the system, and prior knowledge of the potential energy landscape. This task is particularly challenging for floppy molecules and, generally, large systems. Several general strategies were recently developed to guide the selection and design of vibrational coordinates, drawing from the available pool of known curvilinear and rectilinear coordinates.^33–35^

Due to the diversity of nuclear motions and their dependence on molecular size and bonding topology, no single coordinate system is universally optimal for describing the vibrations of different molecules. One promising approach to improve the effectiveness of a coordinate system involves developing general coordinates parametrized by variables that can be optimized to minimize vibrational couplings or energy levels. Such general coordinates, expressed as linear combinations of normal coordinates,^36,37^ curvilinear coordinates,^38–44^ or as a quadratic function of normal coordinates,^45^ were shown to significantly enhance the accuracy of variational calculations. Despite these developments, the broader application of coordinate optimization in variational calculations remains largely unexplored, with previous efforts generally limited to linear parametrizations specific to particular systems.

In this work, we introduce a new general nonlinear parametrization for vibrational coordinates that is based on normalizing flows,^46^ implemented using a neural network. The parameters of the neural network are optimized by using the variational principle. Applied to the calculation of the 100 lowest vibrational states in H_2_S, H_2_CO, and HCN/HNC molecules, the present approach achieves several orders of magnitude greater accuracy in energy predictions compared with commonly used curvilinear coordinates for the same number of basis functions. The optimized vibrational coordinates effectively capture the underlying physics of the problem, reduce couplings between different vibrational modes, and remain consistent across various levels of basis set truncation. Building on this property, we propose a cost-efficient approach in which the coordinates are first optimized by using a small number of basis functions and then applied to calculations with a larger number of basis functions, keeping the parameters of the neural network fixed.

## II Methods

### II.I Enhancing a Basis Set by Change of Coordinates

We begin by choosing a truncated set of orthonormal basis functions {ϕ_*n*_}_*n* = 0_^*N*^ of *L* ^2^ along with an invertible map *g*_θ_ parametrized by a set of parameters θ. *g*_θ_ maps an initial set of vibrational coordinates **r** to a new set of coordinates **q** of the same dimension, i.e., **q** = *g*_θ_(**r**). To improve the approximation properties of the basis functions ϕ_*n*_, we evaluate them in **q** to obtain a new set of augmented basis functions {γ_*n*_(**q**; θ)}_*n* = 0_^*N*^ defined as

1 $$\gamma_{n}\left(q;\theta \right)≔\phi_{n}\left(g_{\theta }\left(r\right)\right)\sqrt{\left|\det\nabla_{r}g_{\theta }\left(r\right)\right|}$$

where multiplying by the square root of the determinant of the Jacobian ensures that the basis functions remain orthonormal with respect to the *L* ^2^-inner product in the vibrational coordinates, independent of the values of θ. Inducing augmented basis functions by a nonlinear change of variables is analogous to inducing an augmented probability distribution *p* from a base distribution *p*_0_ using a change of variables *g*_θ_, commonly referred to as a normalizing flow in the machine learning literature.^46,47^ Therefore, we refer to *g*_θ_ as a normalizing flow and to **q** as a normalizing-flow coordinate.

The map *g*_θ_ can, in principle, be any differentiable invertible function. However, to maintain the completeness of the augmented basis set {γ_*n*_(**q**; θ)}_*n* = 0_^∞^, the normalizing flow must have a non-zero derivative.^48^ We construct *g*_θ_ using an invertible residual neural network (iResNet).^49^ We refer the readers to the Supporting Information for a more detailed explanation of the equivalence between optimizing basis sets and vibrational coordinates.

### II.II Architecture

By construction, iResNet, which is commonly used for image processing, places no restrictions on the output domain. However, in many computational physics and chemistry applications, the domain of internal coordinates is inherently bounded. For example, in vibrational calculations, internal coordinates often represent distances, which are strictly positive, or angles, which are typically periodic or confined to a finite interval such as [0, π] or [0, 2π]. Mapping into nonphysical or redundant ranges of internal coordinates can lead to inaccurate numerical outcomes and violations of the variational limit. For example, the iResNet output may extend into coordinate regions where the potential is undefined, leading to unreliable results.

To address these issues, we developed an invertible flow that enabled control over the output ranges. First, we mapped the outer-most quadrature points of the basis sets in each dimension to −1 and 1 using a fixed linear scaling. This way, we allow any domain of the primitive basis set to be handled identically. Next, iResNet was applied, producing an unbounded output. To map the output back to a finite interval, we applied a wrapper function, *L*. This wrapper function can be any mapping that transforms an infinite domain into a finite domain. We selected the tanh function to map values to the interval [−1, 1]. Finally, we applied a linear scaling **a**·**x** + **b** to adjust the output to the desired target interval. The scaling parameters **a** and **b** were optimized as part of the workflow. To ensure the output remains within the correct interval, we used a subparametrization of the linear parameters *a*_*i*_(α) and *b*_*i*_(β), where α and β are optimizable variables. We applied different functions for different coordinates depending on their specific range requirements. Three different subparametrizations were used to accommodate infinite, semi-infinite, and finite intervals. A schematic of this workflow is provided in Figure 1.

**Figure 1:**
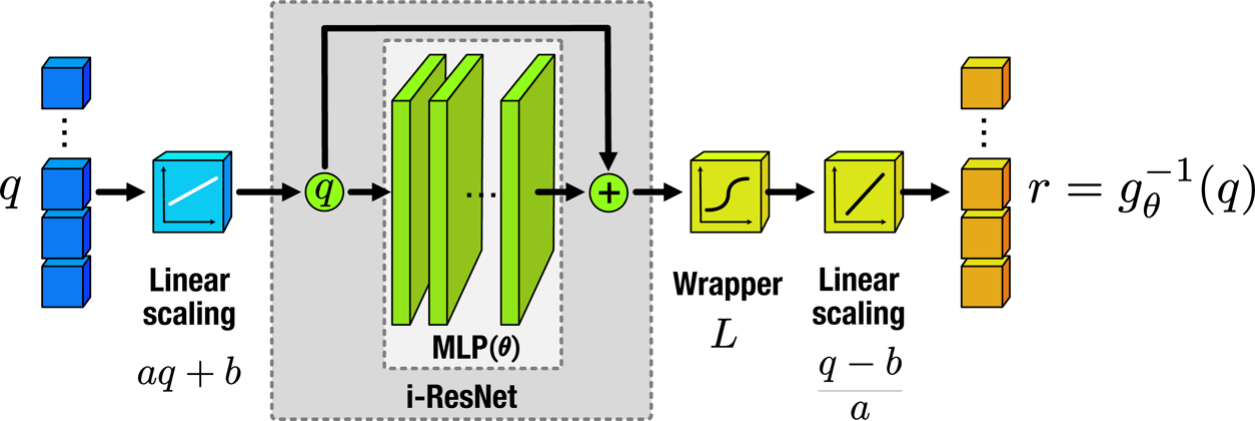
Schematic diagram of the computational workflow of the normalizing-flow function. As wrapping functions, we used *L* = tanh­(*q*). A fixed scaling procedure was applied to map the initial coordinate ranges to an identical domain. The linear scaling parameters *a* and *b* were optimized together with the multilayer perceptron θ-parameters.

### II.III Construction of the Hamiltonian

The matrix elements of the vibrational kinetic and potential energy operators in the augmented basis (eq 1) can be expressed by introducing a change of variables **q** = *g*_θ_(**r**) into the integrals. For the potential, this results in the following expression.

2 $$V_{n\prime n}=\left\langle \gamma_{n\prime }\right|V\left|\gamma_{n}\right\rangle =\int \phi_{n\prime }^{*}\left(q\right)V\left(g_{\theta }^{-1}\left(q\right)\right)\phi_{n}\left(q\right)dq$$

This illustrates that the normalizing flow *g*_θ_ effectively modifies the coordinates in which the operators are expressed for a fixed set of basis functions {ϕ_*n*_}_*n*_.

The matrix elements of the kinetic energy operator in the augmented basis set are given by

3 $$\begin{array}{l} T_{\mathrm{nn}\prime }=\int \phi_{n}^{*}\left(q\right)\mathrm{T̂}\left(g_{\theta }^{-1}\left(q\right)\right)\phi_{n\prime }\left(q\right)dq\\ =\frac{\hbar^{2}}{2}\sum_{\mathrm{kl}}\int \left[\left(\frac{1}{2\sqrt{D}}\frac{\partial D}{\partial q_{k}}+\sqrt{D}\frac{\partial }{\partial q_{k}}\right)\phi_{n}^{*}\left(q\right)\right]\\ \times \sum_{\lambda \mu }\frac{\partial q_{k}}{\partial r_{\lambda }}G_{\lambda \mu }\left(g_{\theta }^{-1}\left(q\right)\right)\frac{\partial q_{l}}{\partial r_{\mu }}\\ \times \left[\left(\frac{1}{2\sqrt{D}}\frac{\partial D}{\partial q_{l}}+\sqrt{D}\frac{\partial }{\partial q_{l}}\right)\phi_{n\prime }\left(q\right)\right]dq \end{array}$$

*D* = |1/det ∇_**q**_ *g*_θ_^–1^(**q**)|, *G*_λμ_ is the kinetic energy matrix, λ and μ indices are used to denote the elements of the coordinate vector **r**, and *k* and *l* indices denote the elements of the coordinate vector **q**, i.e., *q*_*k*_ = *g*_θ,*k*_(**r**) and *r*_λ_ = *g*_θ,λ_^–1^(**q**). The differential operators, $\frac{\partial }{\partial q_{k}}$ , only operate inside the square brackets. To obtain this formula, we employed integration by parts, enabling the second-order derivative operator to act symmetrically on both the bra and the ket functions as first-order derivatives. The boundary term is omitted, as its contribution is zero for most integration domains. Additionally, the kinetic energy operator includes the so-called pseudopotential term, which originates from the transformation from Cartesian to the initial internal coordinates. It is calculated as

4 $$U=\frac{\hbar^{2}}{32}\sum_{\lambda }\sum_{\mu }\frac{G_{\lambda \mu }}{{\mathrm{g̃}}^{2}}\frac{\partial \mathrm{g̃}}{\partial r_{\lambda }}\frac{\partial \mathrm{g̃}}{\partial r_{\mu }}+4\frac{\partial }{\partial r_{\lambda }}\left(\frac{G_{\lambda \mu }}{\mathrm{g̃}}\frac{\partial \mathrm{g̃}}{\partial r_{\mu }}\right)$$

where *g̃* = det­(*G* ^–1^). The pseudopotential is a scalar operator, and its matrix elements in the transformed basis can be expressed analogously to the potential energy matrix elements in (eq 2).

### II.IV Optimization

We approximate the vibrational wave functions Ψ_*m*_ (*m* = 1,..., *M*) as a linear combination of augmented basis functions (eq 1), i.e.,

5 $$\Psi_{m}\left(q\right)\approx \sum_{n\leq N}c_{\mathrm{nm}}\gamma_{n}\left(q;\theta \right)$$

The linear-expansion coefficients *c*_*nm*_ and the normalizing-flow parameters θ are determined using the variational principle by minimizing the energies of the ground and excited vibrational states. For the coefficients, this is equivalent to solving the eigenvalue problem **E** = **C** ^–1^ **HC**, where **C** = {*c*_*nm*_}_*n*,*m*_^*N*,*M*^, **E** = {*E*_*m*_}_*m*_^*M*^ are the vibrational energies, and **H** = **T** + **V** + **U** is the sum of matrix representations of the kinetic, potential, and pseudo potential energy operators, given by eqs 2–4.

Because vibrational energies are nonlinear functions of the parameters θ, these parameters are optimized by using gradient descent methods. The optimization is guided by a loss function derived from the variational principle and may involve minimizing quantities such as the sum of vibrational energies, the trace of the Hamiltonian matrix, or the matrix exponential. A loss function expressed as the sum of all energies spanned by the chosen basis set is equivalent to the trace of the Hamiltonian matrix,

6 $$L_{\theta }=\sum_{n\leq N}E_{n}=\mathrm{Tr}\left(H\right)\to \min_{\theta }$$

This loss function has a relatively low computational cost, as it decouples the nonlinear parameters θ from the eigenvector coefficients *c*_*nm*_, requiring only the evaluation of diagonal elements of the Hamiltonian matrix when the initial basis is orthonormal. In contrast, when the loss function is based on the sum of a subset of the lowest energies, the parameters depend on the eigenvector coefficients *c*_*nm*_, and repeated solutions of the eigenvalue problem during optimization are required. Despite the added complexity, the high accuracy achieved, even with a small number of basis functions, can potentially outweigh the computational costs of the repeated matrix diagonalization. In our calculations, we used the sum of a subset of all vibrational energies as the loss function, *vide infra*. A cost-efficient optimization and application strategy that mitigates the cost of repeated matrix diagonalization is discussed in Section 3.4.

The evaluation of the matrix elements in eqs 2–4 is one of the most computationally demanding parts. In this work, we employed Gaussian quadratures to compute the necessary integrals, altering the quadrature degree in different optimization steps to prevent overfitting. We found that alternating between smaller quadratures during optimization was computationally more efficient while still converging to the same values of the parameters θ as those obtained using a larger quadrature. After convergence, the final energies and wave functions were computed by solving the eigenvalue problem with a large quadrature for accurate integral evaluations. For higher-dimensional systems, more efficient techniques such as sparse-grid methods^50^ or collocation^51^ can be used. Alternatively, Monte Carlo methods^52,53^ may be employed when high accuracy is not required.

### II.V Computational Details

The accuracy and performance of our approach were validated in calculations of vibrational states for hydrogen sulfide H_2_S, formaldehyde H_2_CO, and hydrogen cyanide/hydrogen isocyanide HCN/HNC isomers. For H_2_S and H_2_CO, we used valence coordinates as the reference coordinates and employed a direct product of the Hermite functions as the basis set. For H_2_S, the direct product basis was constructed by considering only combinations of one-dimensional (1D) vibrational quantum numbers (*n*_1_, *n*_2_, *n*_3_) that satisfy the polyad condition 2*n*_1_ + 2*n*_2_ + *n*_3_ ≤ *P*_max_, where *n*_1_, *n*_2_, and *n*_3_ correspond to the vibrational quanta for *r*_SH_1__, *r*_SH_2__, and α_∠H_1_SH_2__ valence coordinates, respectively. For H_2_CO, we applied the basis truncation condition 2*n*_1_ + 2*n*_2_ + 2*n*_3_ + *n*_4_ + *n*_5_ + *n*_6_ ≤ *P*_max_, where *n*_1_,..., *n*_6_ correspond to the vibrational quanta for valence coordinates *r*_CO_, *r*_CH_1__, *r*_CH_2__, α_∠OCH_1__, α_∠OCH_2__, and τ, the dihedral angle between the OCH_1_ and OCH_2_ planes. For HCN/HNC, we used the basis truncation condition 2*n*_1_ + 2*n*_2_ + *n*_3_ ≤ *P*_max_ and Jacobi reference coordinates *r*_CN_, *R*, α_∠R‑CN_, where *R* is the distance between the hydrogen atom and the center of mass of the C–N bond and α_∠R‑CN_ is the angle between these coordinate vectors. Hermite functions were used for the two radial coordinates, and Legendre functions were used for the angular coordinate. The Legendre functions were multiplied by sin^1/2^(α_∠R‑CN_) to ensure the correct behavior of the wave function at the linear geometry of the molecule, where the Hamiltonian becomes singular.^54,55^ For all molecules, we employed spectroscopically refined potential energy surfaces (PES)^56–58^ and numerically constructed exact kinetic energy operator using the method described in refs 13 and 24 .

To model the normalizing flow *g*_θ_, we used an iResNet consisting of 10 blocks. Each block was represented by a dense neural network comprising two hidden layers with unit sizes of [8, 8] and an output layer of *n* units, where *n* corresponds to the number of coordinates. A more detailed description of the iResNet architecture is available in the Supporting Information. The normalizing-flow parameters were optimized variationally by minimizing the sum of the 100 or 200 lowest vibrational energies. Generally, 1000 iterations were enough to achieve good convergence. Benchmark energies were computed with basis sets truncated at *P*_max_ = 60 (optimized for *P*_max_ = 12) for H_2_S, *P*_max_ = 16 (optimized for *P*_max_ = 9) for H_2_CO, and *P*_max_ = 44 for HCN/HNC.

## III Results

### III.I Computed Vibrational Energies

On average, over the 100 lowest energies, the calculations converged with an accuracy of 0.04 cm^–1^ for H_2_S, 0.53 cm^–1^ for H_2_CO, and 0.03 cm^–1^ for HCN/HNC compared to the reference values reported in the literature.^56–58^ We thus considered our results to be converged and used them as benchmark data throughout the rest of the manuscript. A table summarizing the deviations of vibrational energies from the reference values is provided in the Supporting Information.

The absolute error for the 100 lowest vibrational states of H_2_S, H_2_CO, and HCN/HNC, as a function of the basis set truncation parameter *P*_max_, is shown in Figure 2. For each molecule, the results of two variational calculations are presented, one using reference valence or Jacobi coordinates and another using the optimized normalizing-flow coordinates. With the same number of basis functions, coordinate optimization resulted in up to 5 orders of magnitude improvement in the accuracy of vibrational energy calculations compared to using the standard reference coordinates. Extrapolating to a larger number of basis functions, we estimate that matching the same accuracy using the reference coordinates would require approximately an order of magnitude increase in the number of basis functions.

**Figure 2:**
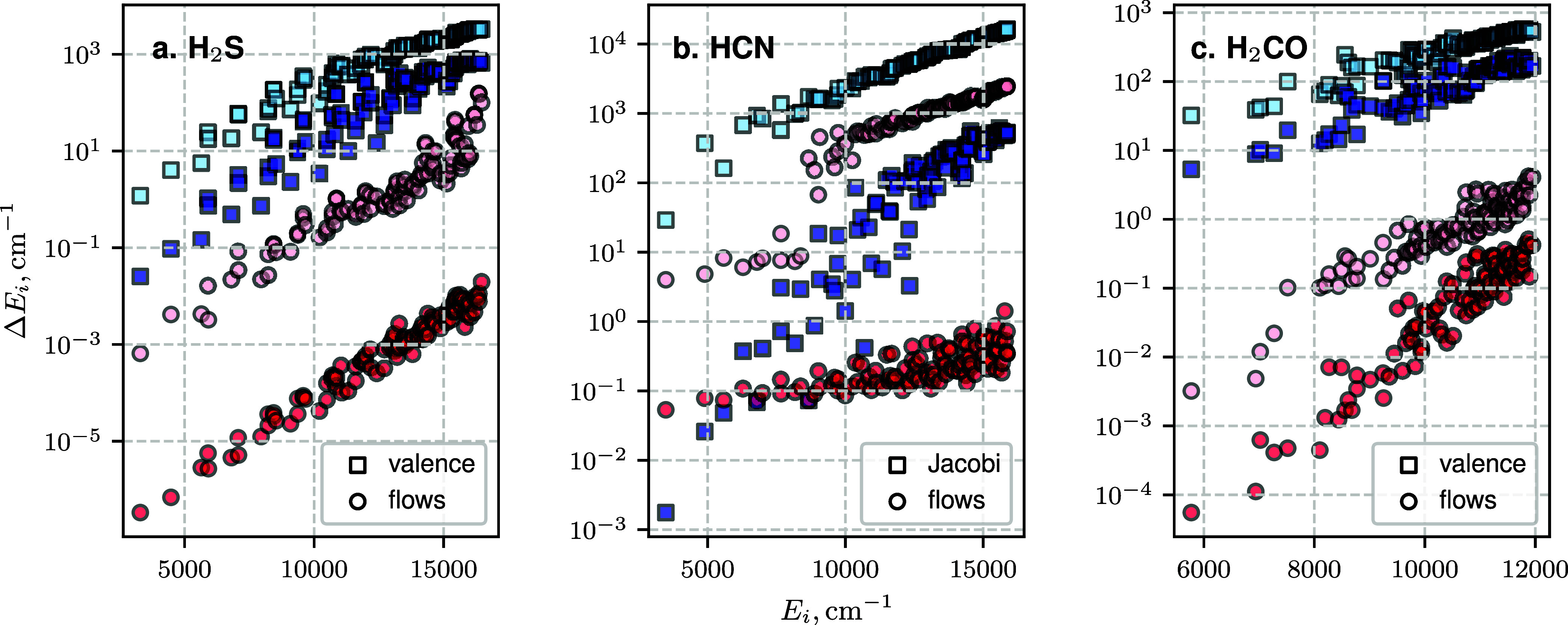
Convergence of H_2_S, HCN, and H_2_CO vibrational energy levels. Plotted are the discrepancies of the 100 lowest energy levels using standard (blue squares) and normalizing-flow (red circles) coordinates. Light and dark colors represent truncations corresponding to a smaller and larger number of basis functions, respectively. (a) Energy discrepancies for H_2_S at *P*_max_ = 12 (140 basis functions) and 20 (506). (b) Energy discrepancies for HCN at *P*_max_ = 12 (140) and 32 (1785). (c) Energy discrepancies for H_2_CO at *P*_max_ = 9 (1176) and 12 (3906).

We note that the convergence of results can also be improved by increasing the complexity of the normalizing-flow function. A detailed analysis of this effect, along with an investigation of how varying the number of target states affects the normalizing-flow coordinates, is provided in the Supporting Information.

Direct product basis sets can be improved using basis set contraction, which involves partitioning the total Hamiltonian into subsystems, solving reduced-dimensional variational problems for each, and then using these solutions for the full-dimensional problem.^59,60^ For molecules such as H_2_S and H_2_CO, basis set contraction works well due to the near-separability of valence coordinates in the PES. However, this approach becomes more challenging for floppy molecules such as HCN/HNC, where two of the Jacobi coordinates are strongly coupled.

This challenge is illustrated in Figure 3 for the first 200 vibrational energies of HCN/HNC. The figure presents results of basis set contraction using Jacobi coordinates alongside those obtained with a direct product basis set of primitive functions, i.e., Hermite and Legendre, using optimized normalizing-flow coordinates. The contracted basis was constructed by partitioning the Hamiltonian into 1D subsystems for each of the Jacobi coordinates, with the HCN isomer equilibrium geometry as the reference configuration. As shown in Figure 3, the contracted basis significantly improves convergence compared to the product basis set results in Figure 2b but not for all vibrational states. High accuracy is achieved only for states localized around the HCN minimum of the PES, while delocalized states and states localized around the HNC minimum show little improvement. In contrast, the optimized normalizing-flow coordinates provide a balanced description of all localized and delocalized states, with a much smaller spread in errors across different states.

**Figure 3:**
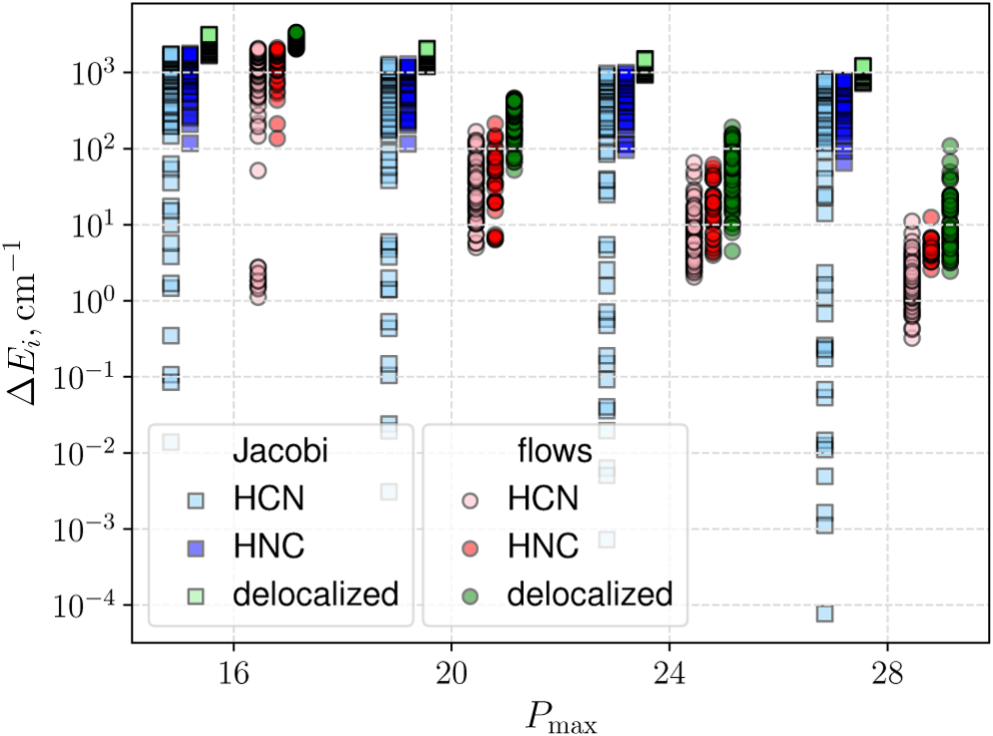
Convergence of HCN/HNC vibrational energy levels. Shown are the lowest 200 energies for using Jacobi coordinates (squares) and normalizing-flow coordinates (circles). The energy discrepancies (Δ*E*_*i*_) relative to our converged benchmark reference are shown for several basis sets, truncated at *P*_max_ = 12 (140 basis functions), 16 (285), 20 (506), 24 (811), and 28 (1200). Vibrational states assigned to the HCN isomer, the HNC isomer, and states with an energy above the isomerization barrier (delocalized) are differentiated by color. All states are slightly offset along the *P*_max_ axis for visual clarity.

### III.II Interpretability

To gain insight into the interpretation of the optimized normalizing-flow coordinates, we plotted in Figure 4 the two-dimensional cut of the PES of HCN/HNC along the strongly coupled Jacobi coordinates, *R* and α_∠R‑CN_, i.e., *V*(*r*_0_, *R*, α_∠R‑CN_), alongside the corresponding cut in the optimized normalizing-flow coordinates, *V*(*g*_θ_^–1^(*q*_1_, *q*_2_, *q*_3_)). In these plots, the *r*_CN_ coordinate is fixed at its equilibrium value *r*_0_ and *q*_1_ = 0. The potential is clearly highly anisotropic when expressed in Jacobi coordinates (panel a), which leads to a strong coupling between the two vibrations. In contrast, when expressed in the optimized coordinates (panel b), the HCN ↔ HNC minimum energy isomerization pathway is practically a straight line along the coordinate *q*_3_ at *q*_2_ ≈ 0. This reduction in anisotropy explains why coordinate optimization improves convergence on the product basis. The optimization achieves an effective coordinate decoupling of the PES, which allows for a better approximation of the eigenfunctions of the Hamiltonian by the chosen direct product basis. In addition, it is evident from the spacing between the contour lines along the flow coordinate *q*_2_ that the potential becomes more harmonic in this dimension in comparison to *R* in the Jacobi coordinates. The same behavior was observed when comparing *q*_1_ and *r*_CN_. This is expected, as Hermite functions, the solutions of the quantum harmonic oscillator, were used as the basis for stretching coordinates.

**Figure 4:**
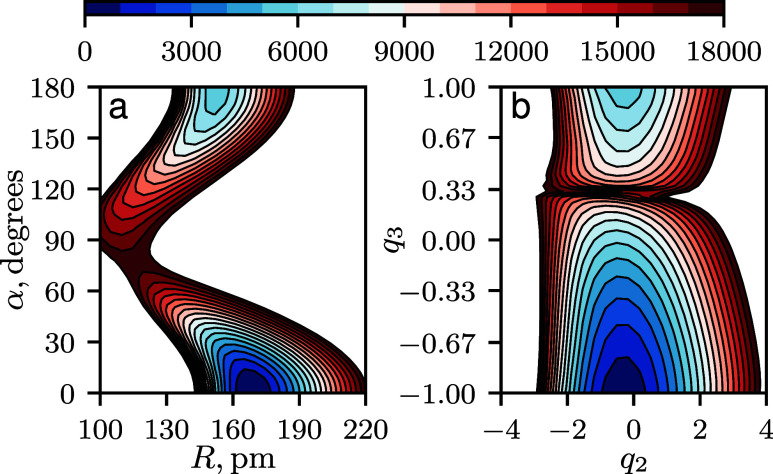
Two-dimensional cuts of the HCN/HNC potential energy surface. (a) Cut along the Jacobi coordinates *R* and α_∠R‑CN_. (b) Cut along the optimized normalizing-flow coordinates. The optimization was performed for a basis set truncated at *P*_max_ = 16 (285 basis functions), with the loss function defined as the sum of the 100 lowest vibrational state energies. An effective decoupling of the surface in the normalizing-flow coordinates is patent.

### III.III Assignment of Approximate Quantum Numbers

Assigning approximate quantum numbers to computed eigenstates connects numerical results to their spectroscopic interpretation. Typically, as the complexity of the method for solving the Schrödinger equation increases, so does the difficulty of assignment. In many cases, less accurate but more interpretable effective models are more practical than highly accurate methods as they facilitate approximate quantum number assignment and enhance the interpretability of experimental spectra.

The enhanced coordinate decoupling in the HCN/HNC Hamiltonian suggests that assigning approximate quantum numbers to computed eigenstates is more straightforward in normalizing-flow coordinates compared to reference Jacobi coordinates. In Table 1, we compare the accuracy of the projection-based assignment of approximate quantum numbers for the first 100 eigenstates of HCN/HNC. Projections were performed onto one-dimensional eigenfunctions (a contracted basis) expressed in either Jacobi or normalizing-flow coordinates. In the limit of convergence of both the three- and one-dimensional eigenfunctions, the projection-based assignment depends only on the choice of coordinates and not on the basis used to compute the eigenfunctions. A unique assignment is ensured when the norm-square of the largest absolute projection coefficient is larger than 0.5. The normalizing-flow coordinates were optimized for 100 eigenstates with *P*_max_ = 28.

**Table 1:** Projection-Based Assignment Metrics for Vibrational States of HCN/HNC^a^

measure	median	mean	min	*N*_assign_
Jacobi (HCN)	0.27	0.35	0.05	24
Jacobi (HNC)	0.17	0.25	0.04	15
Jacobi (HCN/HNC)	0.39	0.46	0.12	39
flows (HCN)	0.80	0.79	0.40	97

a The tabulated values are the median, mean, and minimum values of the largest norm-square projection coefficients obtained by projecting the vibrational wave functions for 100 states onto products of one-dimensional eigenfunctions. In the last column, *N*_assign_, the number of vibrational states (out of 100) that could be unambiguously assigned a unique set of approximate quantum numbers are shown.

The contracted basis was constructed by partitioning the Hamiltonian into one-dimensional subsystems corresponding to each coordinate. For Jacobi coordinates, either the HCN or the HNC equilibrium geometry was used as reference configurations. In contrast, for normalizing-flow coordinates, only the HCN equilibrium geometry was needed due to reduced vibrational coupling. The assignment of approximate quantum numbers is significantly more accurate using the normalizing-flow coordinates with 97 uniquely assigned states compared with only 39 using Jacobi coordinates and choosing the largest projection coefficient of the HCN and HNC calculations. This highlights the benefits of optimized coordinate transformations for more reliable spectroscopic interpretations of the results.

### III.IV Transferability

We found that the converged iResNet parameters remained nearly identical when optimized using different basis set truncations, suggesting that unique optimal vibrational coordinates exist for a given type of basis. Leveraging this finding, we developed a cost-efficient approach where the flow coordinates are first optimized using a small number of basis functions and then applied with fixed parameters in calculations with a larger number of basis functions. The size and computational cost of the quantities that depend on the normalizing flow, such as $\frac{\partial q_{\alpha }}{\partial r_{l}}$ , $\frac{\partial D}{\partial r_{l}}$ , $\frac{1}{D}$ , etc., are independent of the number of employed basis functions. This means that calculations using fixed (pretrained) normalizing-flow parameters scale with the truncation parameter, *P*_max_, in the same way as those using a regular linear mapping. The transferability property significantly reduces the computational costs and makes it feasible to apply our approach to high-dimensional systems while maintaining accuracy comparable to full optimization.

In Figure 5, we show the convergence for the 100 lowest energy levels of H_2_S with respect to the basis set truncation parameter, *P*_max_. The results are obtained using valence and optimized normalizing-flow coordinates. Two types of normalizing-flow coordinates are compared: those optimized for each specific *P*_max_ (Opt. flows) and those optimized for selected values of *P*_max_ (12, 16, 20) and subsequently transferred to calculations with larger *P*_max_. The metric for the convergence is the error of the individual energy levels (*E*_*i*_ – *E*_*i*_^(ref)^), where *E*_*i*_^(ref)^ represents the benchmark energies detailed in the Supporting Information. The results clearly demonstrate that energy calculations using transferred coordinates yield greater accuracy than those using valence coordinates. Moreover, their performance is on par with the more computationally intensive Opt. flows coordinates. The findings also indicate that transferring from a larger *P*_max_ can enhance the accuracy of highly excited states.

**Figure 5:**
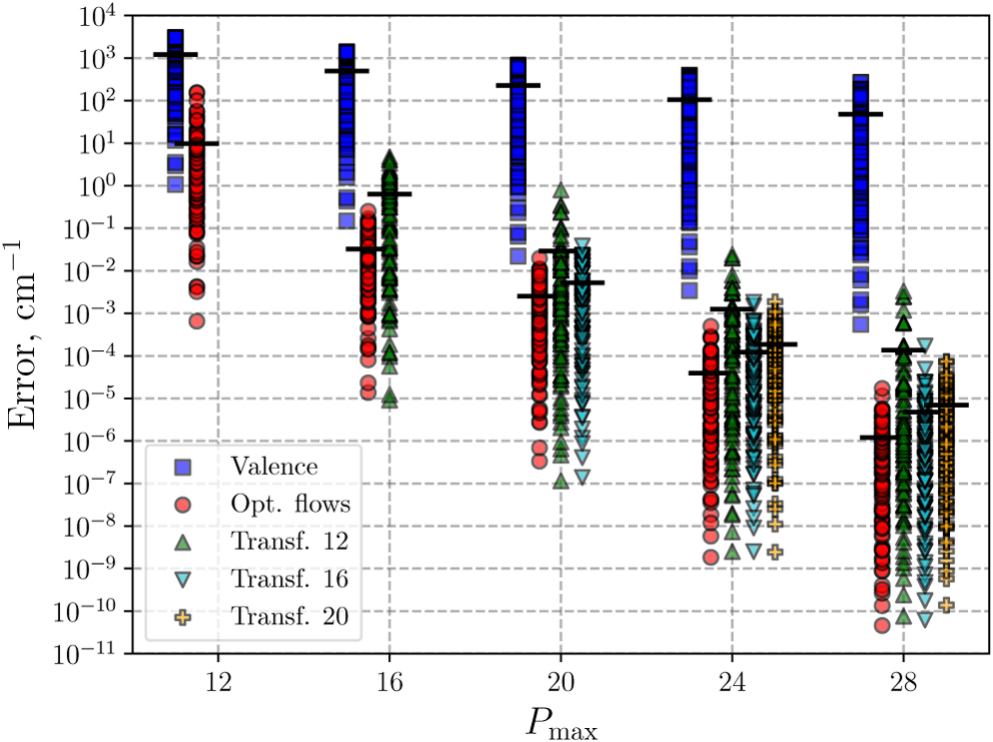
Convergence of the first 100 vibrational energies of H_2_S. Shown are the results obtained for H_2_S using valence (blue squares) and optimized normalizing-flow (red circles) coordinates as a function of *P*_max_. Results obtained with normalizing-flow coordinates optimized for *P*_max_ = 12 (green up-triangles), *P*_max_ = 16 (cyan down-triangles), and *P*_max_ = 20 (orange plus signs) and applied to calculations with larger *P*_max_ are also shown. Thick horizontal lines indicate the average energy-level error for each *P*_max_, which is minimized through training. Data points are slightly offset along the *P*_max_ axis for visual clarity.

The convergence of the approximation using the Hermite basis with respect to the number of basis functions, *N*, is algebraic.^61^ Specifically, the error satisfies

$$∥\Psi_{m}-{\mathrm{\Psi ̂}}_{m}∥<AN^{-k}$$

where ∥·∥ denotes the *L* ^2^-norm, Ψ_*m*_ is the exact wave function, and Ψ̂_*m*_ is its approximation. The constant *A* depends on the relationship between the target wave function and the operators associated with the Hermite basis, and *k* denotes the rate of convergence. The convergence for the Hermite basis defined in normalizing-flow coordinates is also algebraic,^62^ with different constants *A* and *k* for each map. Therefore, it is reasonable to assume that the loss function defined in eq 6 converges algebraically, i.e.,

$$L\left(N\right)=\frac{L_{\theta }^{100}\left(N\right)-L_{\mathrm{Ref}}^{100}}{100}∼AN^{-k}$$

where θ are the optimized parameters for the chosen *N*. To quantify the improved convergence rate observed for the normalizing-flow coordinates (see Figure 5), we fitted log(L) with a linear expression in *N*, i.e., log(L)=−klog(N)+log(A) . The regression parameters derived from this fit are listed in Table 2. The convergence rate of the two normalizing-flow coordinates is significantly higher than that of the valence coordinates. Remarkably, the convergence rate of the transferred normalizing-flow coordinates reaches 75% of the convergence rate of the flow coordinates optimized at each truncation level. The constant *A* is also decreased by the use of nonlinear coordinates, which means that the accuracy is improved for any fixed truncation.

**Table 2:** Convergence Parameters for Different Coordinates

coordinate	*k* × 10^3^	log (*A*)
valence	0.61 ± 0.08	11.7 ± 0.3
opt. flows	2.90 ± 0.47	6.43 ± 2
transf. 12	2.17 ± 0.30	7.11 ± 1

The results for *P*_max_ = 12 for H_2_CO in Figure 2c were calculated with the normalizing-flow coordinate optimized for *P*_max_ = 9. Additional results provided in the Supporting Information further demonstrate the utility of the transferability property. In future work, we will elaborate on the transferability property across basis set truncations and investigate the extension of the principle of transferability of normalizing-flow coordinates to different isotopologues and to molecular systems sharing similar structural motifs. This could potentially contribute to our understanding of intrinsic vibrational coordinates.

## IV Conclusions

In summary, we introduced a general nonlinear parametrization for vibrational coordinates of molecules by using normalizing flows. By optimizing the normalizing-flow parameters through the variational principle, we significantly accelerated basis set convergence, leading to more accurate vibrational energies. The improvement is especially pronounced for highly excited and delocalized vibrational states. The learned coordinates enhanced the separability of the Hamiltonian, which we leveraged to improve the assignment of approximate quantum numbers by projection onto direct products of one-dimensional eigenfunctions. The enhanced separability also potentially allows for a more intuitive interpretation of the key motifs in strongly coupled vibrational dynamics. The transferability of the optimized coordinates across different truncation levels provides a computationally efficient protocol for more complex molecular system calculations. As other variational approaches, our method suffers from exponential growth in the size of the product basis as the number of coordinates increases. This challenge has been effectively addressed in the literature using prescreening techniques that selectively retain only the most relevant basis-product configurations for the states of interest.^63,64^ It should be possible to combine the present normalizing-flow approach with state-specific eigenvalue solvers, where the basis-product configurations are tailored to specific vibrational states. One promising method specifically designed for vibrational solutions is the iterative residuum-based RACE algorithm.^65^ After the release of the first arXiv version of this work,^66^ another group integrated the concept of normalizing flows for basis set augmentation with Monte Carlo methods and successfully applied it to high-dimensional systems.^67^

We also explored the applicability of the normalizing-flow method for excited electronic states, testing it on single-electron systems, such as the hydrogen atom, hydrogen molecular ion, and carbon atom in the single-active electron approximation. Results presented in the Supporting Information show a significant improvement in basis set convergence. This suggests the promising potential of the normalizing-flow method for electronic structure problems, especially since neural network-based methods for excited state computations remain challenging.^53,68^

## Supplementary Material



## Data Availability

The results reported in this manuscript did not depend on any specific data.

## Notes

A detailed mathematical description of the normalizing-flow approach, along with additional details on our computational setup, reference calculations, and further investigations, an application of the normalizing-flow method to excited electronic states (PDF)

## Notes

**Code Availability** The code developed in this work is publicly available at https://gitlab.desy.de/CMI/CMI-public/flows/-/releases/v0.1.0.
