# Supplementary material for: Computing Excited States of Molecules Using Normalizing Flows
Source: J Chem Theory Comput. 2025 May 15;21(10):5221–9. doi: 10.1021/acs.jctc.5c00590 (PMC12120919; doi:10.1021/acs.jctc.5c00590)
Supplement: Supplementary file 1 [file ct5c00590_si_001.pdf]

Supplementary information:
Computing excited states of molecules using normalizing flows

Yahya Saleh,[1,2,*] Álvaro Fernández Corral,[2,3] Emil Vogt,[2]
Armin Iske,[1] Jochen Küpper,[2,3,4] and Andrey Yachmenev[2,4,*]

[1] *Department of Mathematics, Universität Hamburg, Bundesstr. 55, 20146, Hamburg, Germany*
[2] *Center for Free-Electron Laser Science CFEL, Deutsches Elektronen-Synchrotron DESY, Notkestr. 85, 22607 Hamburg, Germany*
[3] *Department of Physics, Universität Hamburg, Luruper Chaussee 149, 22761 Hamburg, Germany*
[4] *Center for Ultrafast Imaging, Universität Hamburg, Luruper Chaussee 149, 22761 Hamburg, Germany*

(Dated: 2025-05-08)

Learning an optimized vibrational coordinate system $\mathbf{r} \to g_\theta(\mathbf{r}) = \mathbf{q}$ and learning a new basis set $\{\phi_n(\mathbf{r})\}_{n=0}^{\infty} \to \{\phi_n(\mathbf{q})\sqrt{|1/\det \nabla_{\mathbf{q}} g_\theta^{-1}(\mathbf{q})|}\}_{n=0}^{\infty}$ are two faces of the same coin, as seen, e. g., from performing a change of variable in the matrix representation of the potential

$$\mathbf{V}_{n'n} = \langle \gamma_{n'} | V | \gamma_n \rangle = \int \phi_{n'}^*(\mathbf{q}) V(g_\theta^{-1}(\mathbf{q})) \phi_n(\mathbf{q}) \mathrm{d}\mathbf{q}.$$

In the following, we give a detailed description of the latter perspective, highlighting theoretical foundations and linking to the concept of normalizing flows in machine learning. We also provide detailed information on the numerical simulations conducted in this study, along with additional results that can faciliate the application of this method in other domains.

I. MATHEMATICAL FOUNDATIONS

Composing a basis set $\{\phi_n(\mathbf{r})\}_{n=0}^{\infty}$ with an invertible mapping g yields a sequence of functions $\{\phi_n(\mathbf{q})\sqrt{|1/\det \nabla_{\mathbf{q}} g^{-1}(\mathbf{q})|}\}_{n=0}^{\infty}$. Certain choices of the mapping g can produce sequences of functions with improved approximation properties [1]. A common example is the linear map $g(x) = a\ x + b$, where a and b are chosen to align the potential of the problem with the potential that generates the basis set around the equilibrium geometry of the molecule. There are also examples of nonlinear fixed mappings, such as trigonometric or exponential functions.

However, the use of adaptive nonlinear mappings introduces additional complexity. To ensure convergence of the method, the basis set must retain its completeness after compositing with the mapping g. The concept of composing basis sets with an invertible neural network was first introduced in Cranmer *et al.* [2]. However, the approach was only applied to simple models, and the rationale for employing invertible neural networks was not fully justified or rigorously explored.

[*] Email: yahya.saleh@cfel.de
[*] Email: andrey.yachmenev@cfel.de;
URL: https://www.controlled-molecule-imaging.org

The rationale for using invertible neural networks from an approximation-theory perspective was first analyzed in Saleh *et al.* [3] and later rigorously established in Saleh and Iske [4], where sufficient and necessary conditions for completeness under perturbation by composition operators were characterized.

Normalizing flows are foundational models in the field of generative machine learning [5], rooted in the probability integral transform initially introduced in Fisher [6]. This theorem states that any continuous probability density p can be converted by a change of variables to a uniform distribution on the interval $[0, 1]$. By imposing a few reasonable conditions to p, e. g., finiteness, it can be shown that this change of variables is bijective. As a result, any pair of probability densities p and q that satisfy these conditions can be related through a change of variables, as both can be bijectively mapped into the uniform distribution.

To illustrate this concept, consider the following example, which is commonly used for its simplicity. Let p be an unknown bimodal probability distribution and consider approximating it by the Gaussian distribution p_0. The Gaussian distribution is a simple distribution characterized by only two parameters, the mean and the variance. However, this approximation of p *via* p_0 is inherently flawed since no combination of the mean and the variance can make the Gaussian p_0 exhibit a bimodal behavior. However, as discussed earlier, we can introduce an invertible change of variables $q = g(x)$ which allows us to transform the Gaussian distribution p_0 into a more complex distribution p^A that can approximate p. Specifically, we define the new distribution p^A as

$$p^A(x) = p_0(g(x))|\det \nabla_x g(x)|.$$

Multiplying by $|\det \nabla_x g(x)|$, the determinant of the Jacobian of the change of variables, ensures that the distribution remains normalized. Given that both the Gaussian and bimodal distributions are well-behaved, there exists a suitable function g that can bridge this gap. In this case, we parametrize g as an invertible function g_θ, thus augmenting the expressivity of the simple Gaussian model. The results can be found in FIG. S1.

If the expressivity of a single Gaussian distribution can be enhanced, why not extend this approach to a family

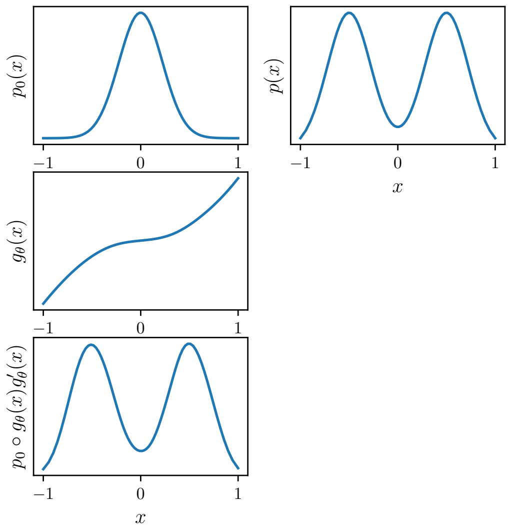

Figure S1. An illustration of normalizing flows augmenting a Gaussian distribution p_0. Approximating a bimodal distribution p with a single Gaussian is not possible. However, composing p_0 with the function g_θ produces a good approximation.

of functions, such as a basis set? This concept forms a core idea of our manuscript.

II. COMPUTATIONAL DETAILS

A summary of the atomic masses used in this study are provided in TABLE S1.

Table S1. The atomic masses utilized in calculations of the vibrational energies for the H_2S, H_2CO and HCN/HNC molecules.

Atom	Mass (u)
Hydrogen	1.00782505
Sulfur	31.97207070
Carbon	12.0
Oxygen	15.99491463
Nitrogen	14.003074004251

A. Architecture of the invertible residual network

In this work, we model the normalizing flow g_θ as an invertible residual neural network (iResNet) [7]. An iResNet is constructed by concatenating blocks of the form

$$\mathbf{x}_{k+1} = \mathbf{x}_k + \mathbf{f}_k(\mathbf{x}_k; \theta),$$

where $\mathbf{f}_k$ is the k-th block, $\mathbf{x}_k$ is the input to this block, and $\mathbf{x}_{k+1}$ is the output. We denote the initial set of coordinates as $\mathbf{x}_0$, and for an iResNet with K blocks, we denote its output as $\mathbf{q} = \mathbf{x}_{K-1}$. Each residual block is a standard feed-forward neural network composed of weights, biases and nonlinear activations. Each such block is guaranteed to be invertible if $\mathbf{f}_k$ is Lipschitz, with Lipschitz constant < 1. The inverse of each block can be computed by a fixed-point iteration method.

To guarantee that the blocks $\mathbf{f}_k$ for $k = 0, \ldots, K-1$ satisfy the Lipschitz condition, we used the LipSwish activation function

$$\sigma(x) := \left(\frac{1}{1.1}\right) \frac{1}{1 + \exp(-x)},$$

which has a Lipschitz constant ≈ 1. A block $\mathbf{f}_k$ containing such activation functions is thus guaranteed to be Lipschitz if each of the linear passes has a Lipschitz constant < 1, i. e., the weight matrices W are normalized to have a spectral norm < 1. This is achieved by setting

$$W = \begin{cases} W & \text{if } \|W\|_2 < c \\ c \cdot \frac{W}{\|W\|_2} & \text{if } \|W\|_2 \geq c, \end{cases}$$

where $0 < c < 1$ is a hyperparameter and $\|W\|_2$ is the spectral norm of the matrix W. For a block $\mathbf{f}_k$ containing n hidden layers, it can be shown that

$$\text{Lip}(\mathbf{f}_k) \leq c^{n+1}.$$

A special attention must be given to the architecture of each block. While a block with a Lipschitz constant close to 1 allows for a greater flexibility, a higher Lipschitz constant can significantly hinder the convergence of the fixed-point iteration method used for computing its inverse. To balance these effects, we used blocks with 2 hidden layers for all of our calculations and set $c = 0.9$, producing blocks with Lipschitz constants ~ 0.7.

B. Comparison against reference data

Benchmark results for vibrational energy calculations were generated using the wavefunction ansatz described in Eq. 3 of the manuscript. The calculations employed a basis polyad truncation $P_{\max} = 60$ for H_2S, $P_{\max} = 16$ for H_2CO, and $P_{\max} = 44$ and for HCN/HCN. We compared our results with those of Azzam *et al.* [8] for H_2S, Al-Refaie *et al.* [9] for H_2CO, and Van Mourik *et al.* [10] for HCN/HNC.

In TABLE S2, we present the calculated energies E_i, the reference values E_i^{ref} and the deviations $\Delta E_i = E_i - E_i^{\text{ref}}$ across the $i = 1..100$ lowest energy levels for the three molecular systems. The results demonstrate almost exact agreement with the reference data, with most of the states converging to even lower limits than the reference data.

Table S2: Comparison of vibrational energies (in cm^{-1}) of H_2S, HCN/HNC, and H_2CO molecules, calculated using the normalizing-flow approach E_i, with reference energies E_i^{ref} [8–10], across the 100 lowest vibrational states. A positive deviation ΔE_i indicates a better convergence is achieved by our prediction.

i	**H_2S**			**HCN**			**H_2CO**		
	E_i	E_i^{ref}	ΔE_i	E_i	E_i^{ref}	ΔE_i	E_i	E_i^{ref}	ΔE_i
1	0.00	0.00	0.00	0.00	0.00	0.00	0.00	0.00	0.00
2	1182.57	1182.58	-0.01	1414.92	1414.92	-0.00	1167.29	1167.34	-0.05
3	2353.91	2353.96	-0.06	2100.58	2100.58	0.00	1249.06	1249.07	-0.01
4	2614.39	2614.41	-0.01	2801.46	2801.46	-0.00	1500.10	1500.12	-0.03
5	2628.46	2628.45	0.01	3307.74	3307.75	-0.01	1746.03	1746.05	-0.02
6	3513.70	3513.79	-0.09	3510.99	3510.99	0.00	2327.34	2327.50	-0.15
7	3779.19	3779.17	0.02	4176.24	4176.24	0.00	2422.56	2422.63	-0.07
8	3789.27	3789.27	0.00	4181.46	4181.45	0.01	2494.25	2494.32	-0.07
9	4661.61	4661.67	-0.07	4686.28	4686.29	-0.01	2666.81	2667.04	-0.22
10	4932.69	4932.70	-0.01	4891.76	4891.76	0.00	2718.97	2719.08	-0.12
11	4939.13	4939.10	0.03	5185.57	5185.64	-0.07	2782.37	2782.41	-0.04
12	5145.03	5144.99	0.05	5394.43	5394.43	0.00	2843.32	2843.34	-0.01
13	5147.17	5147.22	-0.05	5537.76	5537.76	-0.00	2905.75	2905.86	-0.11
14	5243.16	5243.10	0.06	5586.50	5586.50	0.00	2998.91	2999.01	-0.10
15	5797.21	5797.23	-0.03	6033.72	6033.72	-0.00	2999.96	3000.00	-0.04
16	6074.57	6074.58	-0.02	6127.51	6127.56	-0.05	3238.84	3238.94	-0.10
17	6077.63	6077.59	0.03	6242.43	6242.42	0.00	3471.66	3471.72	-0.06
18	6288.13	6288.15	-0.01	6260.59	6260.59	-0.00	3480.98	3481.30	-0.31
19	6289.13	6289.17	-0.04	6513.48	6513.50	-0.02	3585.63	3585.89	-0.26
20	6385.32	6385.32	-0.00	6768.51	6768.51	-0.00	3675.03	3675.21	-0.18
21	6920.08	6920.08	-0.00	6879.60	6879.60	0.00	3736.63	3737.05	-0.42
22	7204.31	7204.31	-0.00	6960.99	6960.99	-0.00	3825.37	3825.97	-0.60
23	7204.43	7204.44	-0.00	7088.71	7088.74	-0.03	3887.05	3887.36	-0.31
24	7419.85	7419.85	-0.00	7210.51	7210.59	-0.08	3935.85	3936.44	-0.59
25	7420.08	7420.09	-0.02	7369.18	7369.18	-0.00	3941.30	3941.53	-0.23
26	7516.83	7516.83	-0.00	7461.59	7461.60	-0.01	3996.26	3996.48	-0.23
27	7576.41	7576.38	0.03	7617.24	7617.24	0.00	4022.46	4022.56	-0.09
28	7576.60	7576.54	0.05	7641.28	7641.28	0.00	4057.79	4058.10	-0.31
29	7752.34	7752.26	0.08	7855.83	7855.84	-0.01	4083.44	4083.49	-0.05
30	7779.35	7779.32	0.03	8020.41	8020.45	-0.04	4163.89	4164.13	-0.24
31	8029.81	8029.81	-0.00	8110.25	8110.25	-0.00	4164.61	4165.27	-0.66
32	8318.68	8318.68	-0.00	8140.64	8140.69	-0.05	4192.77	4193.19	-0.41
33	8321.86	8321.86	-0.00	8197.55	8197.55	-0.00	4247.40	4247.61	-0.21
34	8539.57	8539.56	0.00	8283.37	8283.37	0.00	4256.12	4256.31	-0.19
35	8539.82	8539.82	-0.00	8323.52	8323.52	-0.00	4335.01	4335.11	-0.10
36	8637.16	8637.16	-0.00	8584.71	8584.74	-0.03	4397.64	4398.13	-0.49
37	8697.13	8697.14	-0.01	8691.66	8691.67	-0.01	4466.76	4467.12	-0.35
38	8697.18	8697.16	0.02	8830.27	8830.27	-0.00	4495.16	4495.50	-0.34
39	8878.59	8878.59	-0.00	8850.65	8850.74	-0.09	4529.49	4529.64	-0.15
40	8897.38	8897.38	-0.00	8945.48	8945.50	-0.02	4571.53	4571.66	-0.12
41	9126.09	9126.09	-0.00	8954.46	8954.47	-0.01	4623.65	4623.93	-0.28
42	9420.24	9420.24	-0.00	9009.00	9009.00	0.00	4628.67	4629.22	-0.55
43	9426.39	9426.39	-0.00	9088.01	9088.05	-0.04	4729.67	4730.04	-0.37
44	9647.10	9647.17	-0.07	9164.06	9164.08	-0.02	4734.14	4734.33	-0.19
45	9647.61	9647.61	-0.00	9214.77	9214.85	-0.08	4740.89	4741.40	-0.52
46	9745.80	9745.80	-0.00	9440.11	9440.12	-0.01	4840.27	4840.80	-0.53
47	9806.71	9806.67	0.05	9488.55	9488.54	0.01	4926.09	4926.53	-0.44
48	9806.75	9806.73	0.01	9508.91	9508.91	-0.00	4956.57	4956.91	-0.34

49	9911.10	9911.02	0.08	9619.20	9619.29	-0.09	4977.12	4978.25	-1.12
50	9911.11	9911.02	0.09	9674.67	9674.67	-0.00	4977.37	4978.44	-1.08
51	9993.68	9993.68	-0.00	9675.25	9675.25	0.00	5041.43	5042.22	-0.78
52	10004.98	10004.98	-0.00	9743.71	9743.79	-0.08	5092.00	5092.58	-0.58
53	10188.36	10188.36	-0.00	9865.30	9865.33	-0.03	5108.70	5109.39	-0.68
54	10194.51	10194.45	0.06	9922.90	9922.92	-0.02	5140.25	5141.01	-0.76
55	10208.77	10208.77	-0.00	9993.94	9993.95	-0.01	5153.10	5154.39	-1.29
56	10292.54	10292.54	-0.00	10006.55	10006.59	-0.04	5177.42	5177.65	-0.24
57	10508.45	10508.45	-0.00	10132.33	10132.38	-0.05	5186.87	5187.25	-0.38
58	10517.59	10517.59	-0.00	10165.62	10165.60	0.02	5204.40	5205.00	-0.61
59	10742.14	10742.14	-0.00	10266.38	10266.40	-0.02	5246.10	5246.39	-0.29
60	10742.73	10742.73	-0.00	10304.11	10304.10	0.01	5255.85	5256.28	-0.44
61	10842.18	10842.18	-0.00	10364.93	10364.90	0.03	5312.30	5312.86	-0.57
62	10904.69	10904.69	-0.00	10460.51	10460.50	0.01	5320.86	5322.42	-1.56
63	10904.77	10904.77	-0.00	10636.68	10636.70	-0.02	5324.95	5325.30	-0.35
64	11008.77	11008.70	0.08	10654.81	10654.87	-0.06	5357.25	5358.09	-0.84
65	11008.79	11008.77	0.01	10749.73	10749.70	0.03	5385.38	5386.43	-1.05
66	11097.17	11097.17	-0.00	10753.95	10753.98	-0.03	5415.13	5415.83	-0.70
67	11101.53	11101.53	-0.00	10758.04	10758.00	0.04	5417.34	5417.91	-0.56
68	11278.08	11278.08	-0.00	10861.98	10862.15	-0.17	5432.57	5433.31	-0.75
69	11291.96	11291.96	-0.00	10871.09	10871.10	-0.01	5462.80	5463.15	-0.35
70	11294.93	11294.93	-0.00	10922.00	10922.03	-0.03	5489.37	5490.04	-0.67
71	11391.68	11391.68	-0.00	10925.18	10925.30	-0.12	5489.92	5490.58	-0.65
72	11582.88	11582.88	-0.00	11006.52	11006.50	0.02	5532.31	5532.38	-0.06
73	11595.14	11595.14	-0.00	11035.70	11035.70	0.00	5543.86	5544.61	-0.75
74	11824.13	11824.13	-0.00	11065.18	11065.23	-0.05	5552.06	5552.88	-0.82
75	11824.65	11824.65	-0.00	11198.41	11198.51	-0.10	5625.66	5626.49	-0.83
76	11925.74	11925.75	-0.00	11225.82	11225.90	-0.08	5651.04	5651.16	-0.13
77	11990.55	11990.55	-0.00	11271.68	11271.70	-0.02	5659.48	5660.99	-1.51
78	11990.65	11990.65	-0.00	11489.39	11489.40	-0.01	5666.66	5667.46	-0.80
79	12095.40	12095.40	-0.00	11536.09	11536.10	-0.01	5680.97	5681.46	-0.50
80	12095.44	12095.44	-0.00	11539.63	11539.71	-0.08	5687.28	5688.23	-0.96
81	12149.52	12149.46	0.06	11549.66	11549.60	0.06	5718.43	5718.83	-0.40
82	12149.55	12149.52	0.04	11589.99	11590.02	-0.03	5731.64	5732.19	-0.55
83	12186.40	12186.40	-0.00	11672.67	11672.80	-0.13	5766.47	5766.91	-0.44
84	12188.55	12188.55	-0.00	11688.05	11688.10	-0.05	5768.41	5769.06	-0.65
85	12334.64	12334.64	-0.00	11710.13	11710.10	0.03	5770.76	5771.67	-0.92
86	12383.69	12383.69	-0.00	11744.32	11744.36	-0.04	5809.52	5809.85	-0.33
87	12384.60	12384.60	-0.00	11744.36	11744.50	-0.14	5822.38	5822.71	-0.33
88	12481.05	12481.05	-0.00	11832.45	11832.49	-0.04	5887.13	5888.44	-1.31
89	12524.83	12524.64	0.20	11969.83	11969.90	-0.07	5887.96	5888.55	-0.59
90	12525.35	12525.21	0.13	11970.23	11970.27	-0.04	5889.15	5890.16	-1.01
91	12643.27	12643.27	-0.00	11977.84	11977.70	0.14	5936.19	5937.06	-0.87
92	12658.92	12658.92	-0.00	12055.72	12055.70	0.02	5984.87	5985.33	-0.46
93	12695.20	12695.20	-0.00	12102.62	12102.69	-0.07	5987.32	5988.05	-0.73
94	12735.21	12735.21	0.00	12192.45	12192.50	-0.05	5996.72	5997.93	-1.21
95	12892.52	12892.52	-0.00	12200.53	12200.60	-0.07	5998.74	5999.46	-0.72
96	12892.83	12892.83	-0.00	12304.49	12304.50	-0.01	6053.12	6053.56	-0.44
97	12995.95	12995.95	-0.00	12311.73	12311.70	0.03	6091.73	6093.56	-1.83
98	13063.69	13063.69	-0.00	12374.87	12375.11	-0.24	6107.28	6108.32	-1.04
99	13063.80	13063.80	-0.00	12382.04	12382.00	0.04	6122.55	6124.52	-1.96
100	13170.37	13170.37	-0.00	12384.74	12384.75	-0.01	6177.20	6178.84	-1.63

III. FURTHER INVESTIGATIONS

A. Convergence with respect to enhancement of normalizing-flow complexity

In spectral methods, the standard approach for improving the accuracy of computed energy levels is to increase the number of basis functions N. However, this can be computationally expensive, with memory costs scaling as N^2 and certain computational tasks, such as diagonalization of the Hamiltonian matrix, scaling up to N^3. The normalizing-flow approach offers a possibility of enhancing the expressivity of the basis functions by creating a more complex mapping, achieved by adding additional blocks. In FIG. S2, we show the sum of the 100 lowest vibrational energies (loss) of the HCN/HNC isomers as a function of the number of blocks in the iResNet model, for a fixed number of basis functions (at $P_{\max} = 16$).

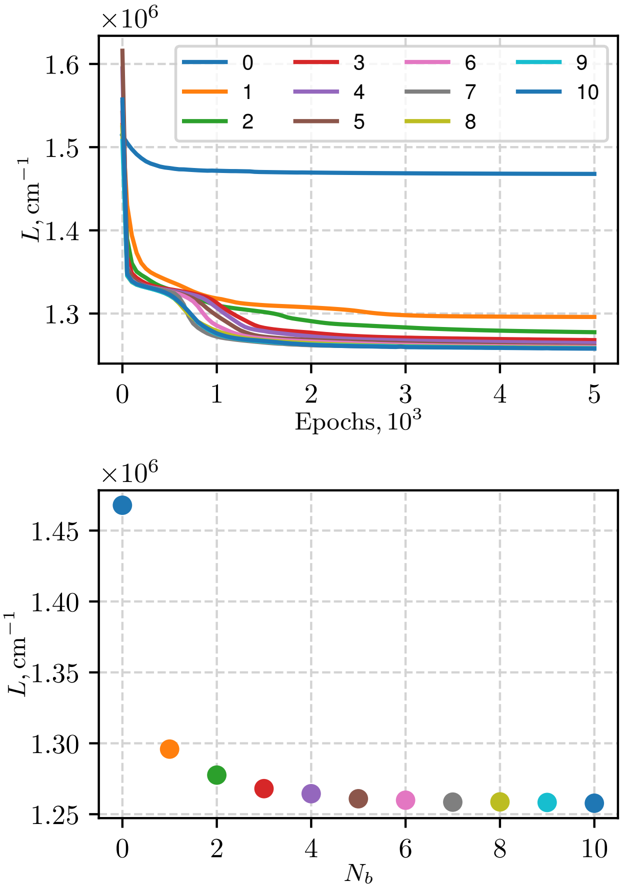

Figure S2. Convergence of the sum of the lowest 100 vibrational energy levels $L = \sum_i^{100} E_i$ for HCN/HNC as a function of the number of blocks in the iResNet model N_b using a basis set truncated at $P_{\max} = 16$. The upper plot shows the convergence of this sum over the number of optimization epochs, with results for different N_b represented by different colors. The lower plot shows the convergence of the optimized sum as a function of N_b.

B. Sensitivity of normalizing-flow coordinates to the number of target states

In this subsection, we examine the impact of optimizing normalizing-flow coordinates for different numbers of target states. In FIG. S3, we show the convergence of the first 200 vibrational energy levels of H_2S, computed with normalizing-flow coordinates with $P_{max} = 20$, corresponding to 506 basis functions. The coordinates were optimized to minimize the lowest 50, 100, 150, and 200 target states, respectively. As shown in FIG. S3, the average error of the first 200 energy levels decreases as the number of target states increases. The decrease in average energy error is accomplished by adjusting the coordinates to better converge the highest energy states in their respective subsets. However, as the number of target states increases, this refinement slightly compromises the convergence of the lowest energy states, which contribute progressively less to the loss function.

C. Transferability of the normalizing flow across different basis set truncation levels

During our investigations into the interpretability of the normalizing-flow coordinates, we noticed that the optimized coordinates remain consistent across different basis set truncations, parametrized by $P_{\max}$. This observation prompted us to explore the transferability of the learned mapping across different truncation levels. Interestingly, we found that a normalizing flow trained with few basis functions could effectively transfer to larger basis sets without the need for retraining. In FIG. S4, we demonstrate this by comparing the vibrational energy levels of HCN/HNC using a normalizing flow initially trained to minimize the sum of the lowest 200 energies with a basis truncated at $P_{\max} = 32$. This mapping was then used with basis sets truncated at $P_{\max} > 32$ and compared to the results of calculations where the normalizing flow was optimized for each truncation level.

The results in FIG. S4 reveal that the transferred normalizing-flow mapping provides comparable accuracy to the optimized mapping for each truncation level and performs significantly better than Jacobi coordinates.

It is worth highlighting that transferability enables significant computational savings. Expanding calculations to incorporate a larger number of basis functions is feasible as long as the quantities dependent on normalizing flow, such as $\frac{\partial q_\alpha}{\partial r_l}$, $\frac{\partial D}{\partial r_l}$, $\frac{1}{D}$, etc, can be efficiently stored in memory or recomputed on the fly. Importantly, the size and computation cost of these quantities are independent of the basis size, meaning that calculations using a pre-

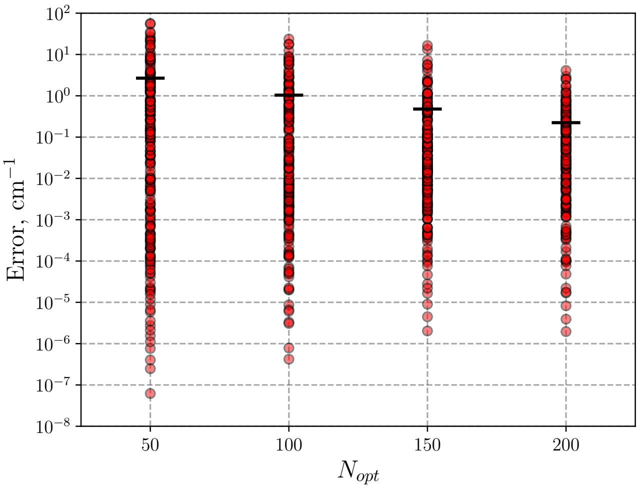

Figure S3. Convergence of the lowest 200 vibrational energy levels ($E_i, i = 1..200$) for H_2S, calculated using $P_{\max} = 20$ (506 basis functions) and normalizing-flow coordinates. The energy discrepancies (ΔE_i) relative to our converged benchmark reference are shown for normalizing-flow coordinates optimized for different numbers of target states (N_{opt}). The solid black lines show the average energy discrepancy.

trained normalizing flow scale with basis size in the same way as those using a regular linear mapping.

Based on these observations, we propose the following efficient protocol for implementing our approach: (i) Train a normalizing flow on a small basis set and store the optimized parameters. (ii) Transfer the learned coordinates to a larger basis set where training would be computationally prohibitive, then solve the eigenvalue problem using the pre-trained normalizing flow to obtain accurate energy levels. This protocol is particularly useful for high dimensional systems, where training costs are significantly higher. The results for H_2S and H_2CO in TABLE S2 where obtained using this protocol, being transferred from the optimization at $P_{max} = 12$ and $P_{max} = 9$ respectively.

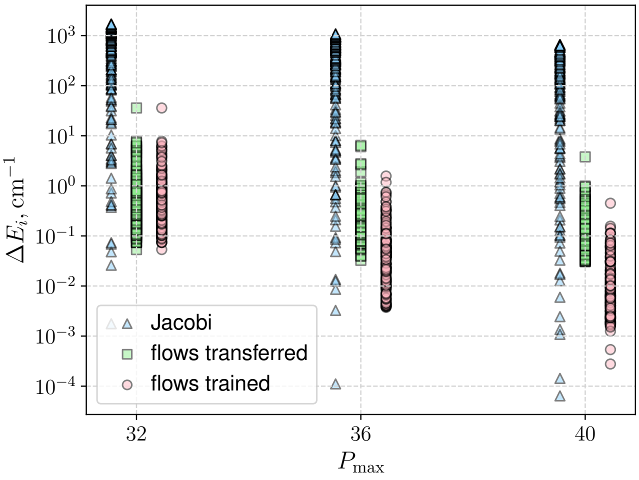

Figure S4. Convergence of the lowest 200 vibrational energy levels ($E_i, i = 1..200$) for HCN/HNC, calculated using Jacobi coordinates (triangles, light blue), normalizing-flow coordinates transferred from $P_{\max} = 32$ (squares, light green), and optimized normalizing-flow coordinates (circles, light pink). The energy discrepancies (ΔE_i) relative to our converged benchmark reference are shown for basis sets truncated at $P_{\max} = 32$ (1785 basis functions), 36 (2470), and 40 (3311). All states are slightly offset along the $P_{\max}$ axis for visual clarity.

D. Electronic calculations

The nonlinear ansatz described in Eq. 3 of the manuscript can be used to solve the electronic Schrödinger equation. To demonstrate this, we computed electronic states of prototypical one-electron systems such as the hydrogen atom, H_2^+ molecular ion, and carbon atom in the single-active electron approximation. We only consider normalizing flows for the radial coordinate and integrated out the angular coordinates using the spherical-harmonic basis. This approach is consistent with common practices in quantum chemistry, where the radial basis typically presents the primary challenge and is the main target of optimization. As a starting basis we explored Hermite basis, the basis of three-dimensional isotropic harmonic oscillator, and the standard atomic-orbital basis sets from Dunning's family [11, 12]. The number of basis functions was defined by the number of radial functions.

The Hamiltonians for the one-electron radial problems were derived by integrating out the angular coordinates using spherical-harmonic functions $Y_{l,m}$ as the angular basis, or solid-harmonic functions $r^l Y_{l,m}$ in the case of atomic-orbital basis sets. For the Hermite and isotropic harmonic oscillator radial basis sets, we utilized a direct product of the radial and angular basis functions with $l \leq l_{\max}$, where $l_{\max} = 3$ for atoms and 8 for molecules. For the atomic-orbital basis set, we employed specific combinations of radial Gaussian and solid-harmonic functions as dictated by the structure of the basis sets.

The angular matrix elements of the electron-nuclei Coulomb-attraction potential were computed using the Laplace expansion, with truncation determined by $l_{\max}$ of the angular basis.

The electronic energies of multi-electron systems, e. g., carbon atom, were calculated using the single-active-electron approximation, in which the electron-electron Coulomb-interaction potential is approximated by the one-electron electrostatic potential created by the electron density $\rho(\mathbf{r})$ of a singly-charged ion in the ground state, i. e.,

$$V(\mathbf{r}) = -\int \frac{\rho(\mathbf{r}')}{|\mathbf{r} - \mathbf{r}'|} d\mathbf{r}'.$$

In addition, the exchange-interaction potential is generally added using some popular approximations, like

the local-density approximation models. Our focus is primarily on investigating the basis set convergence of energies and its enhancement *via* normalizing flows. The absolute accuracy of the electronic energies was not our primary concern, hence we did not include the exchange-interaction potential in our calculations.

In simulations for carbon atom, the one-electron electrostatic potential of C^+ was calculated using the second-order approximate coupled-cluster CC2 level of theory with the aug-cc-pV5Z atomic-orbital basis set [11, 12], as implemented in the Psi4 quantum chemistry package [13]. The calculations were performed on a grid generated by the direct product of an equidistant radial grid $\{r_g\}_g^{N_g}$ and the Lebedev quadrature grid of 131st order represented by a set of angular points $\{\theta_h, \phi_h\}_h^{N_h}$ [14]. The radial potential was calculated by integrating angular coordinates at each radial point in the basis of spherical-harmonic functions using Lebedev quadrature rule, i. e.,

$$V_{l'm',lm}(r_g) = \langle Y_{l'm'}(\theta,\phi)|V(r_g,\theta,\phi)|Y_{lm}(\theta,\phi)\rangle \approx \sum_h^{N_h} w_h Y^*_{l'm'}(\theta_h,\phi_h) Y_{lm}(\theta_h,\phi_h) V(r_g,\theta_h,\phi_h),$$

where w_h is the Lebedev quadrature weight including spherical volume element. For computing the angular integrals between the spherical harmonics centered at different centers, which are needed in atomic-orbital-basis calculations for molecules, we also implemented Becke's partitioning scheme [15]. The calculated values of the radial potential $V_{l'm',lm}(r_g)$ were interpolated across the radial grid $\{r_g\}_g^{N_g}$ using the regular grid interpolator technique.

The reference energies for the hydrogen atom are known analytically, while for the hydrogen-molecule cation H_2^+, the energies calculated using the Riccati-Padé method [16] were employed as the reference. In TABLE S3, we present the absolute errors for the ground and four lowest excited electronic states of H atom and H_2^+, as calculated using normalizing flows with different basis sets. For comparison, the table also includes errors corresponding to full-configuration interaction calculations in large atomic-orbital basis sets aug-cc-pV5Z and aug-cc-pV6Z [11, 12], as computed using the Psi4 quantum chemistry package [13].

The application of the normalizing-flow ansatz with Hermite basis set demonstrates high accuracy and fast basis-set convergence for the radial electronic problem, although such basis is typically considered unsuitable for solving electronic problems. In FIG. S5, we show the convergence of the electronic energies for H atom and H_2^+ molecular ion as a function of the number of Hermite radial basis functions N. The results are compared against the linear parametrization, obtained by setting $g(\mathbf{x}) = \mathbf{a}\cdot\mathbf{x} + \mathbf{b}$ in Eq. 3 of the manuscript, where the linear parameters $\mathbf{a}$ and $\mathbf{b}$ were optimized. Using the normalizing-flow ansatz, the electronic energies of H and H_2^+ converge very quickly to within few mHartree of the

H atom			
State	Hermite	iso-HO	AV6Z
Ground	-4.8	-0.02	-0.0007
1st excited	-0.07	-0.002	-0.08
2d excited	-0.07	-0.002	-1.22
3d excited	-0.07	-0.002	-1.22
4th excited	-0.8	-0.002	-1.22
H_2^+ ion			
State	Hermite	AV5Z	
Ground	-3.1	-0.01	
1st excited	-1.7	-0.01	
2d excited	-0.007	-0.44	
3d excited	-0.007	-0.44	
4th excited	-0.62	-0.53	

Table S3. The errors (exact − calculated, in mHartree) in the ground and excited state calculations for H atom and H_2^+ ($R_{\text{H–H}} = 2$ Bohr), using normalizing flows with Hermite and three-dimensional isotropic harmonic oscillator (iso-HO) basis sets. For comparison, errors corresponding to full-configuration interaction approach with aug-cc-pV6Z (AV6Z) basis for H and aug-cc-pV5Z (AV5Z) basis for H_2^+ are also listed. Note that the normalizing-flow calculations of H_2^+ employed s single-center spherical-harmonic basis truncated at $l_{\max} = 8$, which accounts for estimated $-1.1, -0.2, -10^{-5}, -10^{-5}, -0.15$ mHartree differences in the corresponding electronic energies.

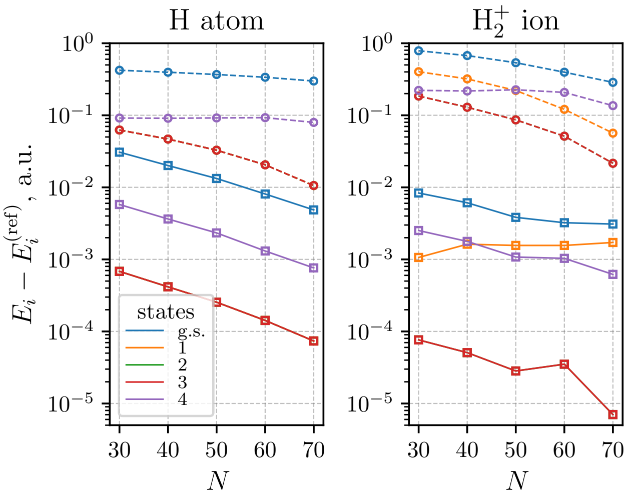

Figure S5. Convergence of the ground (g.s.) and four lowest excited electronic states of hydrogen atom and hydrogen molecular ion H_2^+ ($R_{\text{H–H}} = 2$ Bohr) plotted against the number of Hermite radial basis functions N. Results obtained with optimized-linear (dashed, circles) and normalizing-flow (solid, squares) parameterizations are compared. The reference energies E_i^{ref} for H_2^+ are from Fernández and Garcia [16].

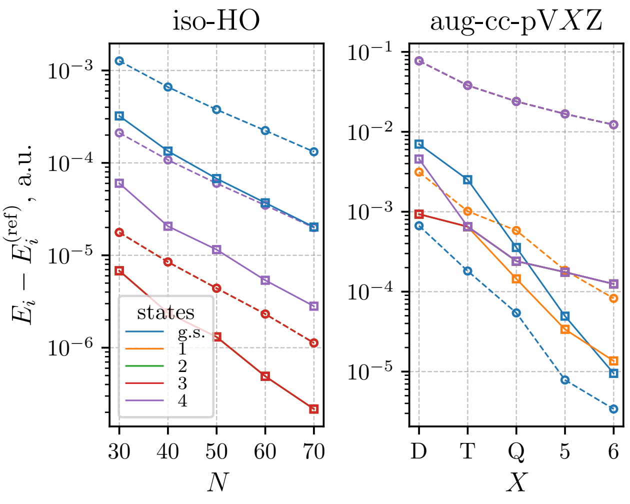

Figure S6. Convergence of the ground (g.s.) and four lowest excited electronic states of hydrogen atom plotted against the size of the radial basis, for the three-dimensional isotropic harmonic oscillator (iso-HO) and atomic-orbital (aug-cc-pVXZ) basis sets. Results obtained with fixed-linear (circles, dashes) and normalizing-flow (solid, squares) parameterizations are compared. Since the four excited states of the hydrogen atom are degenerate, this results in indistinguishable and overlapping error values in some of the plots.

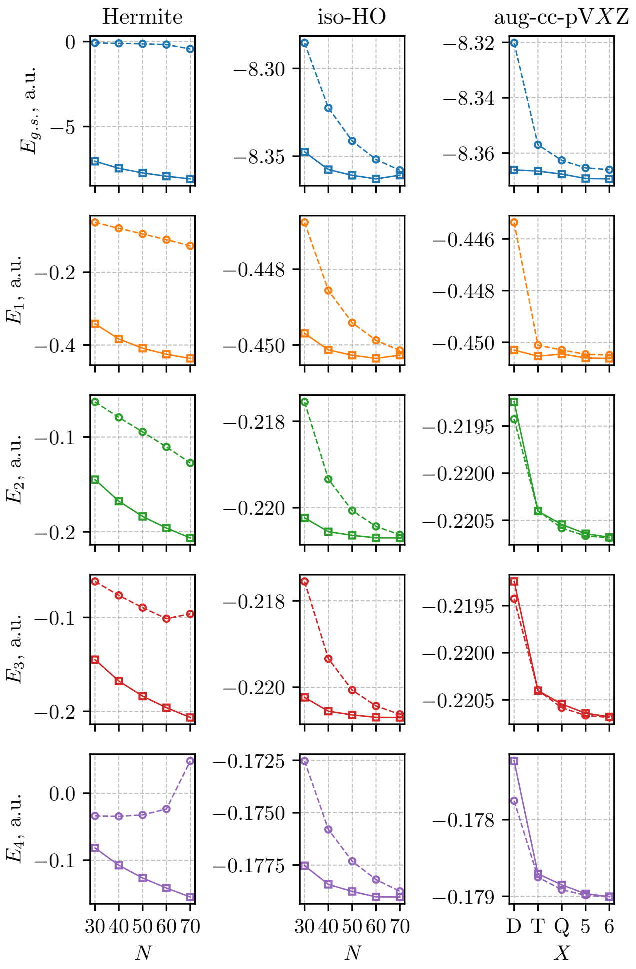

Figure S7. Convergence of the ground (g.s.) and four lowest excited electronic energies of carbon atom plotted against the size of the radial basis N (or X), for the Hermite, three-dimensional isotropic harmonic oscillator (iso-HO) and atomic-orbital (aug-cc-pVXZ) basis sets. Results obtained with optimized-linear (circles, dashed lines) and normalizing-flow (squares, solid lines) parameterizations are compared. The energies are calculated using the single-active-electron approximation with the Coulomb potential obtained from a quantum chemical calculation and neglecting the exchange-interaction potential.

exact values for the ground state, respectively, and even more precisely for the excited states. The accuracy for H_2^+ is constrained to ~ 1 mHartree for the ground state and less than that for excited states due to truncation in the angular basis set. When employing a linear parametrization, a considerably larger number of Hermite-basis functions would be required to reach the same accuracy.

Normalizing flows also improved the performance of more common basis sets used in electronic structure computations, such as the basis set of isotropic three-dimensional harmonic oscillator (iso-HO) and the augmented correlation-consistent aug-cc-pVXZ (X=D, T, Q, 5, 6) atomic orbital basis sets from Dunning's family [11, 12]. Results for hydrogen atom are presented in FIG. S6, illustrating that the iso-HO basis set, while inherently well-suited and fast-converging for the problem, still exhibits a notable enhancement in accuracy when composed with a normalizing flow. Although the original atomic-orbital basis set demonstrates quicker ground-state convergence compared to that composed with a normalizing flow, the latter yields results that are more accurate and fast-converging for excited states. The lack of improvement in the ground-state energy by normalizing flow is likely due to the nature of atomic orbital basis sets, which consist of Gaussian functions with exponents carefully optimized for the ground state of an atom. This specific optimization likely accounts for ineffectiveness of normalizing flow in improving the ground electronic energy.

In FIG. S7, we show the convergence for the ground and four lowest excited electronic states of carbon atom with the number of radial basis functions, as calculated using the normalizing-flow approach and an optimized-linear parametrization. The convergence is plotted for different basis sets, including the Hermite basis set, the isotropic three-dimensional harmonic oscillator basis set, and augmented correlation-consistent atomic orbital basis sets. The data clearly shows that the normalizing-flow approach generally improves the accuracy of results for all states across different basis sets, compared to optimized-linear mapping. However, an exception can be noted in the case of atomic-orbital basis sets. While normalizing-flow parametrization leads to faster convergence for the ground and first excited states, it exhibits comparative

or slightly inferior accuracy for the remaining excited states, particularly with smaller basis sets, as compared to an optimized-linear mapping. We also noticed some convergence issues of the training procedure with a larger number of basis functions, which can be attributed to the limitations in the accuracy of numerical integration with Hermite and Laguerre quadratures used for calculations across all basis sets.

REFERENCES

[1] D. Gottlieb and S. A. Orszag, *Numerical analysis of spectral methods: theory and applications* (SIAM, 1977).

[2] K. Cranmer, S. Golkar, and D. Pappadopulo, Inferring the quantum density matrix with machine learning, arXiv preprint arXiv:1904.05903 (2019), arXiv:1904.05903 [physics].

[3] Y. Saleh, A. Iske, A. Yachmenev, and J. Küpper, Augmenting basis sets by normalizing flows, Proc. Appl. Math. Mech. **23**, e202200239 (2023), arXiv:2212.01383 [math].

[4] Y. Saleh and A. Iske, Inducing Riesz and orthonormal bases in L^2 via composition operators, preprint (2024), arXiv:2406.18613 [math].

[5] G. Papamakarios, E. Nalisnick, D. J. Rezende, S. Mohamed, and B. Lakshminarayanan, Normalizing flows for probabilistic modeling and inference, J. Mach. Learn. Res. **22**, 2617 (2022).

[6] R. Fisher, *Statistical Methods for Research Workers*, Biological monographs and manuals (Oliver and Boyd, 1925).

[7] J. Behrmann, W. Grathwohl, R. T. Q. Chen, D. Duvenaud, and J.-H. Jacobsen, Invertible residual networks, in *Proceedings of the 36th International Conference on Machine Learning*, Proceedings of Machine Learning Research, Vol. 97, edited by K. Chaudhuri and R. Salakhutdinov (PMLR, 2019) pp. 573–582.

[8] A. A. A. Azzam, J. Tennyson, S. N. Yurchenko, and O. V. Naumenko, ExoMol molecular line lists – XVI. the rotation-vibration spectrum of hot H_2S, Mon. Not. R. Astron. Soc. **460**, 4063 (2016).

[9] A. F. Al-Refaie, A. Yachmenev, J. Tennyson, and S. N. Yurchenko, ExoMol line lists – VIII. a variationally computed line list for hot formaldehyde, Mon. Not. R. Astron. Soc. **448**, 1704 (2015).

[10] T. Van Mourik, G. J. Harris, O. L. Polyansky, J. Tennyson, A. G. Császár, and P. J. Knowles, Ab initio global potential, dipole, adiabatic, and relativistic correction surfaces for the hcn–hnc system, J. Chem. Phys. **115**, 3706 (2001).

[11] T. H. Dunning, Gaussian basis sets for use in correlated molecular calculations. I. The atoms boron through neon and hydrogen, J. Chem. Phys. **90**, 1007 (1989).

[12] R. A. Kendall, T. H. Dunning, Jr., and R. J. Harrison, Electron affinities of the first-row atoms revisited. Systematic basis sets and wave functions, J. Chem. Phys. **96**, 6796 (1992).

[13] D. G. A. Smith, L. A. Burns, A. C. Simmonett, R. M. Parrish, M. C. Schieber, R. Galvelis, P. Kraus, H. Kruse, R. D. Remigio, A. Alenaizan, A. M. James, S. Lehtola, J. P. Misiewicz, M. Scheurer, R. A. Shaw, J. B. Schriber, Y. Xie, Z. L. Glick, D. A. Sirianni, J. S. O'Brien, J. M. Waldrop, A. Kumar, E. G. Hohenstein, B. P. Pritchard, B. R. Brooks, H. F. Schaefer, A. Y. Sokolov, K. Patkowski, A. E. DePrince, U. Bozkaya, R. A. King, F. A. Evangelista, J. M. Turney, T. D. Crawford, and C. D. Sherrill, Psi 1.4: Open-source software for high-throughput quantum chemistry, J. Chem. Phys. **152**, 184108 (2020).

[14] V. Lebedev and D. Laikov, A quadrature formula for the sphere of the 131st algebraic order of accuracy, Dokl. Math. **59**, 477 (1999).

[15] A. D. Becke, A multicenter numerical integration scheme for polyatomic molecules, J. Chem. Phys. **88**, 2547–2553 (1988).

[16] F. M. Fernández and J. Garcia, Highly accurate potential energy curves for the hydrogen molecular ion, ChemistrySelect **6**, 9527–9534 (2021).
